# Editorial: Latest breakthroughs in microbial electrochemistry research

**DOI:** 10.3389/fmicb.2022.1100272

**Published:** 2022-12-13

**Authors:** Sebastià Puig, Lluís Bañeras, Annemiek Ter Heijne, Catarina M. Paquete

**Affiliations:** ^1^LEQUIA, Institute of the Environment, University of Girona, Girona, Spain; ^2^Group of Molecular Microbial Ecology, Institute of Aquatic Ecology, University of Girona, Girona, Spain; ^3^Department of Environmental Technology, Wageningen University and Research, Wageningen, Netherlands; ^4^Instituto de Tecnologia Química e Biológica António Xavier, Universidade Nova de Lisboa, Oeiras, Portugal

**Keywords:** extracellular electron transfer (EET), Microbial Electrochemical Technologies (MET), electroactive organisms, microbial electrosynthesis (MES), *Shewanella oneidensis* MR-1, *Sporomusa ovata*

Microbial Electrochemical Technologies (METs) are emerging as promising sustainable solutions to the interconnected problems of population growth and energy poverty while reducing greenhouse gas emissions responsible for climate change. In METs electroactive organisms with the capacity to perform extracellular electron transfer (EET) processes, either oxidize organic matter available in wastes (e.g., wastewaters) and produce current by transferring electrons to an electrode, or receive electrons from the electrode and convert them into useful commodities. Over the last few years, METs have seen significant developments and nowadays they can be used for numerous applications, including electricity generation, wastewater treatment, desalination, bioremediation, biosensing, and the production of biofuels and chemicals, including short chain volatile fatty acids and biofuels (i.e., methane). To expand the application of METs, as well as advance their practical implementation, researchers have focused on the understanding of the EET mechanisms, in the optimization of electrode materials and bioreactor design, as well as in the use of synthetic biology tools to tailor electroactive organisms. In this special issue, the latest breakthroughs regarding these technologies are described, focusing on contributions from the European conference of the International Society for Microbial Electrochemistry and Technology (EU-ISMET2021) held online between 13th and 15th September 2021.

Different bioreactor devices are being currently used for METs applications. Among these bioreactors, electrified biotrickling filters represent one type of device for the sustainable treatment of organic carbon-deficient ammonium-contaminated waters. In Korth et al., the microbiome used on a granule bed cathode in an electrified biotrickling filter has been investigated in relation to reactor performance. Toward this, electrochemical and molecular biological methods were applied, demonstrating that bioelectrochemical denitrification was high within the pH range of 6–10, which allows the use of this type of device in aquaponics systems. Molecular biology analysis showed that a core community exists in the sampled granules, and although typical denitrifiers have been detected in the reactor, none of the species has been previously identified as electroactive.

The application of microbial electrosynthesis (MES) to produce added-value compounds and fuels from CO_2_ are of generally high interest, given its capacity to set the foundations to achieve a circular biobased economy. The predominantly use of obligate anaerobic organisms, in combination with an anode that produces oxygen, creates numerous difficulties that need to be overcome to make this technology scalable. One of these challenges is the protection of the cathode compartment from oxygen since it is the main cause of toxicity and efficiency losses in MES. In Abdollahi et al. this dilemma is presented. In this perspective article, different strategies, including the use of microbial collaboration to scavenge away oxygen, the replacement of the anode reaction, and the purging inert gas in the cathode chamber, are described.

From the organisms that can be used in MES, *Sporomusa ovata* is the biocatalyst with the highest acetate production rate described so far. In Madjarov et al. the research performed on this organism across the different disciplines is reviewed in detail. This review introduces the metabolism used by *S. ovata* and proposes electron uptake mechanisms. Given the importance of this organism in microbial electrosynthesis, this manuscript also describes all the conditions used to grow it on MES for the production of acetate. It outlines the most promising setup, operational condition, and strain, as well as the optimization strategies used so far to advance the practical implementation of this organism in MES.

The low efficiency of EET has led to the development of genetic engineering strategies to start manipulating electroactive organisms. In *Shewanella oneidensis* MR-1, EET is processed by the Mtr pathway, being CctA and FccA the most abundant *c*-type cytochromes in the periplasmic space of this organism. These proteins are responsible for mediating electron transfer from the inner-membrane cytochrome CymA and the porin-cytochrome complex MtrCAB that transfer electrons across the outer membrane. Sun et al. demonstrated that overexpression of CctA increases the electron transfer rate between CymA and MtrCAB, and that NapB, FccA and TsdB in excess impaired EET. This information was used to optimize the composition of periplasmic *c*-type cytochromes to improve EET efficiency. The authors demonstrated that a strain lacking FccA, NapB and TsdB with CctA overexpressed achieved the highest maximum power density in BES, revealing that optimization of the cytochrome profile is a feasible strategy to improve EET efficiency.

Although the number of applications of BES has increased in the last decades, numerous limitations are still required to be overcome to make these systems commercially sustainable. Given the multidisciplinary nature of BES, only with efforts from researchers from different fields (e.g., microbiology, electrochemistry, material science and engineering) it will be possible to find the necessary solutions to advance these systems toward real-world applications, contributing to tackle existing societal challenges.

## Author contributions

All authors listed have made a substantial, direct, and intellectual contribution to the work and approved it for publication.

